# A Novel Triad of Bio-Inspired Design, Digital Fabrication, and Bio-Derived Materials for Personalised Bone Repair

**DOI:** 10.3390/ma17215305

**Published:** 2024-10-31

**Authors:** Greta Dei Rossi, Laura Maria Vergani, Federica Buccino

**Affiliations:** 1 Department of Mechanical Engineering (DMEC), Politecnico di Milano, Via La Masa 1, 20156 Milano, Italy; greta.deirossi@polimi.it (G.D.R.); federica.buccino@polimi.it (F.B.); 2 IRCCS Orthopedic Institute Galeazzi, Via Cristina Belgioioso 173, 20157 Milan, Italy

**Keywords:** bone non-union repair, bio-derived materials, bio-inspired design, digital fabrication, scaffold

## Abstract

The emerging paradigm of personalised bone repair embodies a transformative triad comprising bio-inspired design, digital fabrication, and the exploration of innovative materials. The increasing average age of the population, alongside the rising incidence of fractures associated with age-related conditions such as osteoporosis, necessitates the development of customised, efficient, and minimally invasive treatment modalities as alternatives to conventional methods (e.g., autografts, allografts, Ilizarov distraction, and bone fixators) typically employed to promote bone regeneration. A promising innovative technique involves the use of cellularised scaffolds incorporating mesenchymal stem cells (MSCs). The selection of materials—ranging from metals and ceramics to synthetic or natural bio-derived polymers—combined with a design inspired by natural sources (including bone, corals, algae, shells, silk, and plants) facilitates the replication of geometries, architectures, porosities, biodegradation capabilities, and mechanical properties conducive to physiological bone regeneration. To mimic internal structures and geometries for construct customisation, scaffolds can be designed using Computer-aided Design (CAD) and fabricated via 3D-printing techniques. This approach not only enables precise control over external shapes and internal architectures but also accommodates the use of diverse materials that improve biological performance and provide economic advantages. Finally, advanced numerical models are employed to simulate, analyse, and optimise the complex processes involved in personalised bone regeneration, with computational predictions validated against experimental data and in vivo studies to ascertain the model’s ability to predict the recovery of bone shape and function.

## 1. Introduction

The investigation into bone fractures and subsequent bone regeneration processes is pivotal in the medical field. Crucial in mitigating the mounting prevalence of critical-sized injuries is the identification of fracture-prone regions and the personalisation of bone healing procedures. Advances in this area hold the potential to augment the diagnosis, treatment, and prevention of bone fractures, ultimately leading to enhanced clinical outcomes and an improved quality of life for patients [1,2].

**Figure 1:**
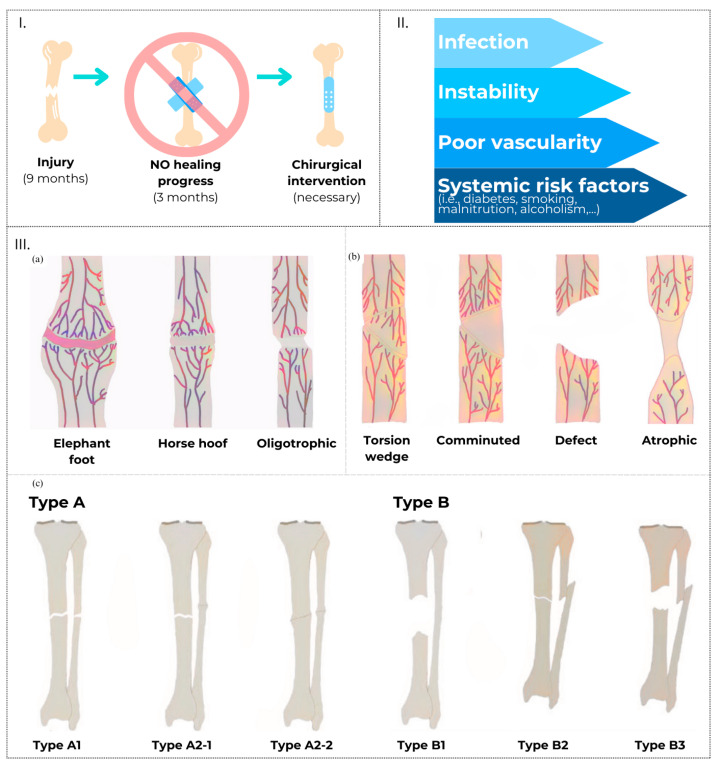
Non-union fractures: definition (**I**) [3,4], causes (**II**) [3,4,11], and classification (**III**) [22,23]. The Weber and Çech classification of hypervascular non-unions (**III-a**) and avascular non-unions (**III-b**). The Paley classification of non-unions (**III-c**) Type A (biologically active) includes hypertrophic non-unions (A1) and oligotrophic non-unions (A2-1 with good contact and A2-2 with poor contact); Type B (biologically inactive) includes necrotic non-unions (B1) and non-unions with segmental bone loss (B2 with minimal loss and B3 with significant loss). The image (**III**) is adapted from [24], under a CC BY-NC 3.0 license (http://creativecommons.org/licenses/by-nc/3.0/, accessed on 15 July 2024). Colours and layout have been modified from the original.

However, bone non-union, a debilitating condition resulting from fractures, infections, instability, tumours, or revision arthroplasty, poses significant challenges (Figure 1I,II). It can result in disability, diminished quality of life, and an elevated risk of infection, multiple surgeries, and even amputation [3,4]. The incidence of non-union in Europe varies depending on region and population, ranging from 5% to 50% of the total fractures, with an overall incidence of non-union following long bone fractures estimated at 5–10% [5]. Notably, these rates fluctuate based on the fracture site, with higher non-union rates observed in tibial fractures (up to 28%) compared to osteoporal fractures (up to 8%) [6]. Additionally, of particular concern to orthopaedic surgeon are the vertebral fractures, characterised by a diffusive barrier hindering signalling between fracture stumps, exhibiting heightened non-union rates (10% to 30%), necessitating surgical reduction in the inter-stump distance [1,7,8], and the application of a physiological loading to have adequate morphological and functional bone structures [8]. Various factors, including patient age, open fractures, tobacco use, diabetes, poor vascularisation, and osteoporosis, influence the risk of non-union (Figure 1II). In particular, osteoporosis increases the risk of bone non-union fractures due to its adverse impact on bone density and strength. Osteoporosis leads to a reduction in bone mass and microarchitectural deterioration, increasing the fragility of bones and their susceptibility to fractures [9,10]. The impaired bone quality in osteoporotic patients can hinder the normal healing process, increasing the likelihood of non-union [11].

While gender-based discrepancies in non-union frequency lack clarity, women’s elevated susceptibility to fractures, attributable to variables such as osteoporosis, reduced bone dimensions, and estrogen deficiency resulting from menopause, implies an augmented predisposition to non-union. These factors not only amplify the risk of fractures but also potentially hinder optimal bone healing, thereby heightening the propensity for non-union in women [12,13,14,15].

The rise in non-union rates leads to a substantial increase in treatment costs due to the need for hospitalisation, costly, invasive procedures, and post-operative care: in Europe, this is exacerbated by an ageing population and higher osteoporosis prevalence [10]. Indirect costs such as lost productivity and prolonged recovery times further strain healthcare systems [16,17,18]. Furthermore, the social impact of non-union is substantial, as patients experience a loss of productivity and increased healthcare utilisation. This is partly due to the protracted healing period for non-union fractures, which can range from 3 to 24 months, with a weighted average duration of approximately 8.6 months [1,7,19].

Consequently, efforts have been focused on adopting minimally invasive approaches to expedite patient recovery and significantly reduce economic costs [20]. Currently, a range of minimally invasive therapeutic modalities is available for non-union management, with treatments typically chosen based on the disease etiology, the specific characteristics of the lesion, and the presence of comorbidities. These interventions aim to provide novel avenues for addressing non-union, anticipating expedited patient recovery and substantial reductions in economic costs [1], while also optimising the efficacy of materials and techniques through the exploration of innovative and advanced materials [21].

### 1.1. Background: Bone Tissue Regeneration as a Challenging Arena

Bone tissue remains a challenging arena to achieve satisfying functional and structural restoration after damage. This is attributed to the intrinsic complexity of bone tissue, characterised by a multi-scale hierarchical architecture that elicits diverse mechanical responses based on the loading scenario. The imperative for satisfactory restoration is underscored by its pivotal role in proper bone remodelling—a complex process orchestrated by the synergistic actions of osteoblasts, osteoclasts, and osteocytes [25].

**Figure 2:**
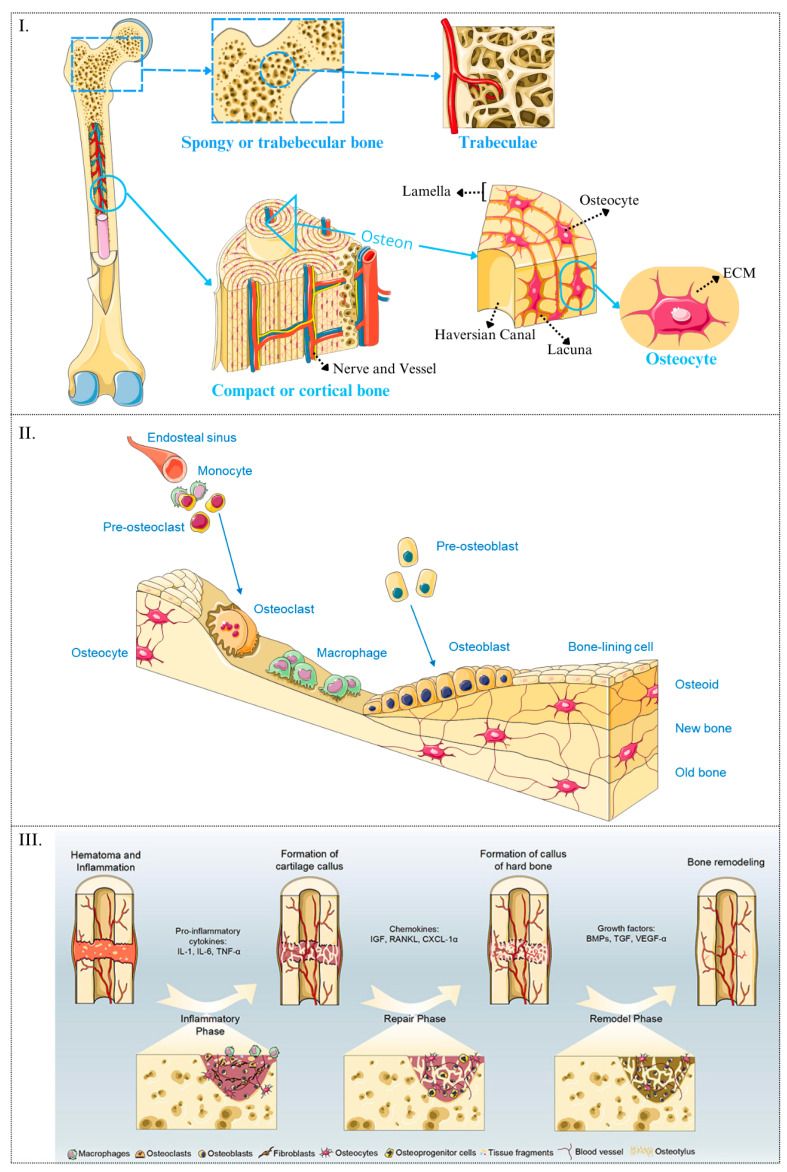
Bone structure is divided into trabecular and cortical bone, each with its respective components (**I**). A schematic of physiological bone remodelling depicts the process of homeostasis, where osteoclasts mediate the removal of damaged bone (osteoclastogenesis) and osteoblasts deposit new matrix (osteogenesis) (**II**). Additionally, a schematic illustrates the four stages of post-fracture bone regeneration, emphasising the cellular components involved and the signalling pathways that regulate these processes (**III**). The image (**III**) is taken from [38], under a CC BY-NC 3.0 license (http://creativecommons.org/licenses/by-nc/3.0/, accessed on 20 October 2024).

Bone, which is a connective tissue in the human body, serves three distinct purposes: mechanical (providing support and protecting organs), metabolic (housing calcium reserves), and homeostatic (harbouring stem cells, including haematopoietic and mesenchymal stem cells, MSCs) functions. The bone extracellular matrix consists of two components—the inorganic (77%) and the organic fraction (23%). The inorganic fraction is adept at conserving calcium, while the organic fraction houses type I collagen and hydroxyapatite crystals synthesised primarily by osteoblasts. In bone tissue, a clear demarcation exists between cortical bone, forming the outer layer, and trabecular or spongy bone, constituting the inner portion of the bone [26]. These two tissue types exhibit distinctive structures: cortical bone has a compact lacune structure and low porosity (approximately 5% to 10%) [27,28], while cancellous bone displays lower density and higher porosity (approximately 50% to 90% [27,28], due to the necessity to accommodate the bone marrow, and playing a pivotal role in blood cell production and immune function [29]) and is distinguished by a network of bony trabeculae (Figure 2I). Additionally, within both tissue types, the presence of lamellar bone tissue is notable, characterised by the orderly arrangement of collagen fibres within the lamellae. Wolff’s law defines bone tissue as adapting its internal architecture in response to mechanical stimuli, thereby optimising its structural integrity to accommodate prevailing mechanical loads [8,25]. Specifically, bone trabeculae orient themselves in accordance with the principal lines of force experienced by the bone. Regions subjected to habitual loading develop trabecular alignment parallel to the direction of force, augmenting the bone’s mechanical competence and enhancing its resistance to mechanical stresses.

Bone remodelling and repair are critical physiological processes that ensure the maintenance of bone strength and structural integrity throughout an individual’s life via tissue homeostasis. Bone remodelling is a multifaceted process initiated by monocytes within the vasculature of the bone marrow. These monocytes differentiate into pre-osteoclasts, which migrate to the bone surface and further differentiate into osteoclasts. Osteoclasts secrete proteolytic enzymes and acids that degrade the mineralised bone matrix, creating resorption lacunae. Concurrently, mesenchymal stem cells (MSCs) within the bone marrow proliferate and differentiate into pre-osteoblasts. These pre-osteoblasts migrate to the resorption sites, where they mature into osteoblasts that synthesise and deposit new bone matrix composed primarily of type I collagen and hydroxyapatite crystals. As the new matrix mineralises, osteoblasts become embedded within it, differentiating into osteocytes. Osteocytes form an extensive network through their dendritic processes, which traverse the mineralised matrix via canaliculi. This cellular network allows osteocytes to sense mechanical loading and communicate with other bone cells, thereby regulating bone remodelling and maintaining bone homeostasis (Figure 2II).

Following trauma, spontaneous bone repair proceeds through two distinct phases: the primary phase and the secondary phase. The primary phase includes the haematoma/inflammatory stage and the granulation tissue formation stage, both regulated by biochemical signals. The secondary phase comprises the formation of the soft callus, the hard callus, and the remodelling stage, which are governed by mechanical cues [30]. The bone tissue exhibits a substantial vascular network, particularly in trabecular bone, which contains numerous blood vessels essential for nutrient delivery and waste removal. Osteocytes, located within the bone matrix, receive nutrients through an extensive network of capillaries and canaliculi, supporting bone metabolism and health [31,32]. Disruption of a long bone leads to blood extravasation and clot formation, initiating an inflammatory response. During this primary phase, platelets release signalling molecules that recruit fibroblasts to the injury site, facilitating the establishment of a provisional collagen matrix and the formation of a new vascular network. In the secondary phase, the initial collagen matrix undergoes chondrogenesis, forming cartilage rich in type I and type II collagen with substantial mechanical properties. This cartilage is subsequently resorbed and replaced by mineralised bone through the coordinated actions of osteoblasts and osteoclasts, ultimately restoring the bone’s structural integrity and supporting the bone marrow [30] (Figure 2III).

The interplay between osteoblasts, osteoclasts, and other cells is crucial for both bone remodelling and repair. Factors such as age, sex, hormones, and environmental conditions can influence these processes, leading to bone loss or fragility. Usually, most bone fractures resolve spontaneously, due to the high regenerative capacity of bone, which is dependent on the age of the subject, the type and severity of the fracture, and the presence or absence of other concomitant pathologies. However, conditions like non-union in critical-sized fractures may hinder bone regeneration (irremediably altering bone remodelling and repair processes), especially in elderly patients with reduced bone mineralisation due to osteoporosis [20]. In non-union fractures, the failure of the initial repair phase is marked by significant stump separation, impeding the diffusion of osteoconductive factors. Scientific evidence suggests that spatial separation of stumps exceeding 2–3 mm results in suboptimal healing outcomes [3,33,34] with thresholds varying for different bone regions (i.e., for clavicle [35] and humerus [36], reportedly extended to 5 mm). The impediment of signalling between stumps leads to a failure of bone regeneration, even in cases of unstable abutments (fracture instability). Furthermore, adequate loading of the bone callus is imperative for successful bone remodelling and the restoration of its initial function and morphology [35,37].

The healing goal for non-union fractures is to achieve structural and functional recovery, restoring the bone to its pre-fracture condition while minimising eventual harm to the patient.

### 1.2. The Existing Gold Standard for Large Bone Injuries Treatment: From Conventional to Regenerative Medicine Strategies

In the literature, bone non-unions are categorised by various characteristics. Notably, the Weber and Çech classification distinguishes between hypervascular non-unions, characterised by an abundant blood supply, and avascular non-unions, which have a poor blood supply. Additionally, the Paley et al. classification further categorises non-unions based on the biological and mechanical environment, providing a comprehensive framework for understanding the underlying pathology. Regardless of type, the ***existing gold standards*** for treating large bone lesions typically involve surgical intervention (Figure 1III). Common treatment modalities include external or internal fixation systems, such as medullary nails, which aim to stabilise the fracture and facilitate osteogenesis by minimising movement at the fracture site [39,40,41]. While this approach allows for self-repair, it inevitably modifies the stress–strain system in the fracture area [39,42]. If an external fixator is utilised, its rigidity enables the maintenance of stability in the fracture lines but also absorbs a significant portion of the load applied to the bone, thereby extending the healing time of the fracture. This occurs because the bone callus is not subjected to physiological stress, potentially slowing down the consolidation process. Conversely, the use of endomedullary fixation nails provides insufficient stability of the fracture lines due to their lower rigidity, resulting in suboptimal healing conditions and limiting the applicability of intramedullary nails.

Advanced surgical techniques, including Ilizarov’s distraction osteogenesis [43,44,45,46,47] and the Masquelet technique [48,49], are also employed. Ilizarov’s technique is a surgical technique used especially where other surgical techniques may prove ineffective; it utilises an external apparatus to apply controlled traction and compression forces to the bone ends, promoting the physiological regenerative capacity of the bone. Despite its efficacy, this procedure is time-intensive (achieving only 1 mm of distraction per day [46]) and can be highly painful for the patient. The Masquelet technique involves the staged removal of nonvital bone tissue, creation of a bone defect, and subsequent grafting with compatible bone, often sourced from allografts (which involve the transplantation of decellularised bone from a transplant bank), to provide a solid base for new bone tissue and promote bone regeneration. Allografts [50,51,52,53] are also used in bone regeneration, enabling the replacement of infected bone tissue with healthy bone tissue from the donor. This technique also offers the advantage of providing a source of viable cells and structural support, which are crucial for the healing process. Unfortunately, its application is predominantly confined to maxillofacial surgery due to the inherent infection risks and the limited availability of suitable donors.

Autograft remains the gold standard for many non-unions, involving the harvest of autologous bone from one site and transplantation to the lesion. This technique, which is also referred to as autologous bone grafting or self-grafting [54,55], is a surgical procedure that uses the patient’s own bone tissue to promote healing and regeneration [53]. This method provides vascularised, healthy bone tissue, enhancing the healing process. Additionally, extracorporeal shock wave therapy (ESWT) [56] offers a non-invasive treatment that can be used alongside autografts. ESWT stimulates angiogenesis and supports bone callus formation by facilitating cell proliferation, thereby further aiding the healing of non-unions and improving clinical outcomes for patients [57]. Despite their biocompatibility and osteogenic properties, autografts cause significant damage at the collection site and are unsuitable for critical-sized lesions.

Each treatment modality presents specific advantages and limitations, highlighting the importance of careful selection and customisation of therapeutic strategies based on the individual patient’s needs and the specific characteristics of the non-union. Therefore, it is necessary to develop ***novel therapeutic materials and surgical therapies***. Bone tissue engineering, particularly the use of scaffolds, shows promise for treating critical-sized lesions by ensuring biomechanical stability, promoting bone osseointegration, and supporting regeneration. Additionally, it stimulates neovascularisation and improves blood supply in the fracture area. In any case, scaffolds constitute a versatile support that can be designed and tailored to fit the patient’s needs and fracture conditions, thereby promoting bone healing and improving clinical outcomes.

The ultimate goal of bone tissue engineering is to generate the ***ideal bone graft*** for improved repair and regeneration. Scaffolds loaded with bone marrow from the iliac crest are considered a promising option for reforming bone tissue [58,59].

However, the use of scaffolds is currently limited to small bone injuries, and their application for critical-sized fractures is still under investigation [60,61,62]. Many pre-clinical and clinical studies have been conducted over the last 30 years to test their effectiveness in facilitating the widespread application of such constructs. These studies highlight an increase in clinical studies related to scaffold utilisation in the last 10 years, especially in Europe, although they are still primarily used for small dental injuries. Concurrently, numerous pre-clinical studies are being conducted to assess their efficacy in non-union fractures of long bones, as this should be the final goal of such constructs. While these studies demonstrate a strong predisposition of scaffolds to promote bone regeneration, much still needs to be studied regarding their ability to accelerate fracture healing time, prevent implant failure, and limit the need for subsequent surgical intervention.

**Figure 3:**
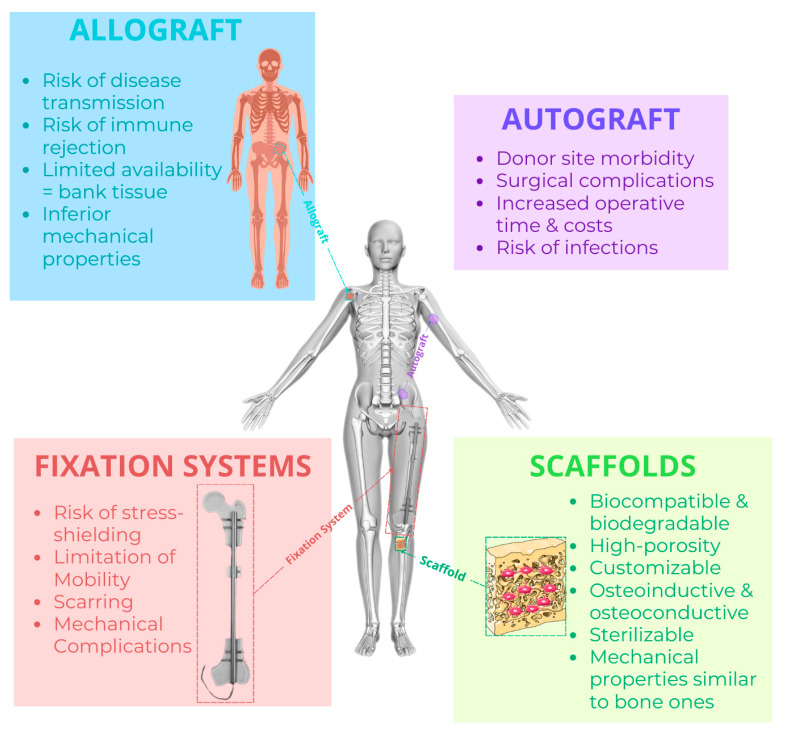
Ideal scaffold characteristics (overcoming conventional therapies). Properties of the ideal bone scaffolds [21,58,59,63,64,65,67,68,69,70,71,72,73,74,75,76,77] in comparison with some disadvantages of the most common bone regeneration treatments (autograft [54,55,77,78], allograft [50,51,52,78], and internal/external fixation systems [39,42]).

The ideal properties of a scaffold for bone regeneration, which overcome the disadvantages of the most common bone fracture treatments, are shown in Figure 3.

Above all, an ideal synthetic scaffold for bone regeneration should mimic the natural extracellular matrix of bone tissue to facilitate cell attachment and proliferation. Its open porous microstructure, with high porosity and pore interconnection, should allow for vascularisation and bone ingrowth, enhancing nutrient and oxygen flow, which are crucial for successful regeneration. The scaffold must also be sterilisable to minimise infection risk, using methods such as autoclaving, gas plasma, ethylene oxide, or UV radiation, depending on the material [63]. Biodegradability ensures controlled degradation and replacement by new bone tissue over time [64], providing a patient-friendly choice because it eliminates the need for a second surgical intervention for implant removal, simultaneously reducing healthcare costs and hospital waste [21,65], which are produced in large quantities, especially during arthroplasty procedures [65,66]. Patient specificity allows scaffolds to be customised in shape and tailored in architecture to meet individual needs [63]. Mechanical properties such as strength, stiffness, and pore size must be considered to provide proper support for growing bone tissue and maintain the ideal mechanical environment for cell differentiation and bone formation. Additionally, scaffolds should be biocompatible, non-immunogenic, and non-toxic. Bioactive and smart scaffolds actively interact with the biological system, promoting efficient bone tissue repair and regeneration. Indeed, these scaffolds are designed to support tissue regeneration by not only providing structural support but also actively interacting with surrounding biological tissues to promote cell attachment, proliferation, and differentiation. The surface topography of the scaffold should promote cell adhesion, proliferation, and differentiation. Finally, a biomimetic and bio-inspired scaffold should mimic the structure and composition of native bone tissue while incorporating biological cues to promote effective tissue repair and regeneration [63].

Some of the properties of ideal scaffolds are also intrinsic to other types of bone regeneration treatments, such as autografts, allografts, and internal/external fixation systems, but none of these bone fracture care types can simultaneously provide all the typical properties of an ideal scaffold (Figure 3).

Regardless of their intrinsic properties, two distinct approaches are employed for scaffold implantation. The first approach involves implanting an acellular scaffold that facilitates the migration, proliferation, and differentiation of native MSCs. The second approach entails pre-seeding the scaffold with MSCs obtained from the patient before implantation. Over the years, numerous materials have been considered for constructing acellular scaffolds suitable for bone regeneration, although the ideal material remains unidentified. Presently, the scaffolds considered fall into two categories: inorganic bone substitutes, including metals, ceramics (especially calcium phosphate ceramics), and bio-glasses; and organic materials, encompassing both synthetic and natural polymers [79,80,81]. The metallic materials employed, such as stainless steel and titanium, are highly biocompatible, cost-effective, and exhibit high resistance. Additionally, magnesium alloys are gaining attention due to their promising properties of biodegradation and the proliferation of osteoblasts facilitated by magnesium hydroxide molecules linked to material corrosion [82,83,84]. Ceramics, notably calcium phosphates like hydroxyapatite, tricalcium phosphate, and biphasic calcium phosphate, are widely used for bone regeneration scaffolds. They boast high biocompatibility and bioactivity, promoting the interaction between ceramics and bone for bonding osteogenesis [82,85,86]. The latest trends in scaffold material selection emphasise natural or synthetic polymers, chosen for their biocompatibility, predictable degradation rate, bioactivity, and capacity to create a biomimetic surface. Prominent among synthetic polymers are polyethylene glycol (PEG) and polyesters, known for their biocompatibility and modifiable degradation rates through copolymerisation. Despite these intriguing properties, many of these materials are non-renewable, as they are derived from fossil-based polymers (i.e., some synthetic polymers) or minerals obtained through mining (i.e., titanium) [87].

Notably, the innovative use of natural polymers, including collagen, alginate, hyaluronic acid, and silk, offers bioactivity, the ability to create biomimetic surfaces, and support for natural remodelling. To improve the field of biomaterial development, it is imperative to introduce synthetic and natural polymers derived from abundant and renewable sources, which offer self-renewability and recyclability, such as polycaprolactone (PCL), polylactic acid (PLA), and poly lactide-co-glycolide (PLGA), or natural fibres [88] and proteins, including silk fibroin [89]. Silk fibroin, in particular, stands out for its cytocompatibility, low immunogenicity, high mechanical resistance, and thermal stability [80,82,90,91].

Simultaneously, studies on bone replacement scaffolds seeded with cells before patient implantation have been conducted. MSCs are commonly used for this regenerative technique, with ceramics being the preferred scaffolds. These scaffolds exhibit superior osteogenic capabilities and greater integration with native tissue compared to acellular scaffolds and have demonstrated efficacy in healing critical-sized lesions.

### 1.3. Current Status and Challenges

Although very promising, bone scaffolds, whether seeded with mesenchymal stem cells before in vivo implantation or not, are fraught with ***several critical issues***. These challenges stem from factors such as the choice of material, scaffold shape, size considerations, and the inherent limitations in controlling the architectural aspects of both the scaffold and the ensuing bone regeneration process. An exhaustive list of these limitations related to diverse materials utilised for scaffold generation is detailed in Table 1 [79,80,81].

Among the common limitations associated with materials typically used for scaffold fabrication, a significant concern is the potential immune response of patients to the materials utilised. Consequently, it is crucial that the selected material demonstrates the highest level of biocompatibility to minimise the risk of chronic inflammation, which could hinder the healing process and ultimately lead to implant failure. Studies have shown that natural materials, such as collagen and silk fibroin, tend to elicit lower immune responses compared to synthetic polymers [92].

To further mitigate immune reactions, scaffolds can be coated with bioactive materials, including hydroxyapatite, which aids in preventing inflammatory responses by releasing growth factors or anti-inflammatory molecules that facilitate improved bone integration [93,94]. Additionally, the biocompatibility and bioactivity of the chosen material are essential for promoting cell adhesion, proliferation, and differentiation—factors critical for effective tissue regeneration.

The biodegradability of bio-derived materials, including collagen, chitin, and both natural and synthetic polymers, also plays a crucial role in bone regeneration [95]. These materials degrade through biological processes involving enzymes and immune cells, breaking them down into simpler components. This degradation—whether hydrolytic, enzymatic, or oxidative—releases growth factors and signalling molecules that can stimulate cellular proliferation and differentiation [96].

Pre-clinical and clinical studies indicate that the use of biodegradable scaffolds can significantly enhance outcomes in bone regeneration [97,98]. For example, materials that gradually release growth factors during degradation have been shown to improve bone healing efficacy in both animal and human models. Conversely, an uncontrolled degradation rate can provide insufficient support for bone growth if too rapid or impede bone integration if too slow. It is also vital to ensure that scaffold degradation within the human body does not provoke an excessive inflammatory response, as this could compromise bone regeneration.

**Table 1:** Limitations related to the various materials used for scaffold implementation.

Materials		Inherent Criticalities	Ref.
**Metals**	**Stainless steel and titanium**	**Biocompatibility**: Stainless steel and titanium are considered biocompatible (with a success rate of 90% in in vivo tests), but could give rise to adverse reactions in the body.	[99]
		**Biodegradability**: Non-biodegradable, i.e., titanium is a non-biodegradable material; it can remain stable in the body for over 20 years without significant degradation.	[100]
		**Mechanical Characteristics**: The rigidity of titanium exceeds that of natural bone tissue. The rigidity of titanium is approximately 110 GPa, compared to 30 GPa of the human bone tissue. The high values of Young’s modulus induce stress shielding.	[93]
		**Other Characteristics**: High thermal conductivity, which can cause damage to surrounding tissues during the application of heat or laser, i.e., the thermal conductivity of stainless steel can range from 15 W/mK to 25 W/mK, while that of titanium can range from 7 W/mK to 22 W/mK.	[101]
		**Imaging**: Can interfere with imaging tests, such as magnetic resonance imaging (MRI) and computerised tomography (CT), making it difficult to assess bone regeneration.	[102]
	**Magnesium alloys**	**Biocompatibility**: No human studies, tested only on animal models.	[103]
		**Biodegradability**: Biodegradable, but the degradation rate depends on the alloy composition and biological environment conditions. Too rapid degradation can cause local inflammation, while too slow degradation can compromise bone regeneration, i.e., the degradation rate of the AZ31 magnesium alloy can be approximately 1.5–2.5 mm/year.	[104]
		**Mechanical Characteristics**: Lower mechanical strength compared to other materials, such as titanium, i.e., the AZ31 magnesium alloy has a tensile strength of approximately 200 MPa.	[104]
		**Other Characteristics**: Prone to rapid corrosion in acidic and saline environments, which can compromise their mechanical strength and stability, i.e., the AZ31 magnesium alloy has a corrosion rate of approximately 0.2 mm/year in physiological solution.	[105]
		**Imaging**: Can interfere with imaging tests, such as magnetic resonance imaging and computerised tomography, due to their low density and high sensitivity to magnetic fields.	[106]
**Ceramics**	**Calcium phosphate ceramics**	**Biocompatibility**: Some calcium phosphate ceramics can cause an inflammatory response in the body, and their biological compatibility depends on the composition of the ceramic and the manufacturing process.	[107,108]
		**Biodegradability**: Gradually degrades in the body, but too rapid degradation can compromise bone regeneration, i.e., the degradation rate of a calcium phosphate ceramic can be approximately 1–3 µm/day.	[109,110]
		**Mechanical Characteristics**: Brittle materials, can easily fracture under mechanical stress. The compressive strength of a calcium phosphate ceramic can be approximately 150–250 MPa.	[111,112]
		**Other Characteristics**: No osteogenic and osteoinductive properties.	[113]
		**Imaging**: Can interfere with imaging tests, such as magnetic resonance imaging (MRI) and computed tomography (CT), due to their high density.	[114]
**Polymers**	**Synthetic: PEG, polyesters, and** **polyurethanes**	**Biocompatibility**: Some of the degradation products may cause immunological reactions and osteolysis without chemical reactions.	[97]
		**Biodegradability**: The degradation rate of synthetic polymers can vary depending on their composition and environmental conditions, i.e., polyethylene glycols (PEG) degrade very slowly and can persist in the body for years, while polyesters such as polylactic acid (PLA) and polyglycolic acid (PGA) can degrade rapidly (6–12 months), but their mechanical stability decreases with degradation.	[115]
		**Mechanical Characteristics**: The mechanical stability and elastic modulus of synthetic polymers can be influenced by their composition and scaffold structure. For example, PLA has an elastic modulus of approximately 2–4 GPa, while PEG has an elastic modulus of approximately 0.1–0.3 GPa.	[116]
		**Other Characteristics**: Polyurethanes have a lower elastic modulus in comparison to native bone, so they are too flexible for load-bearing solutions.	[117]
		**Imaging**: Synthetic polymers can generally be poorly visible in imaging tests, such as magnetic resonance imaging and computed tomography, due to their low density.	[118,119]
	**Natural: collagen, alginate, hyaluronic acid, and silk**	**Biocompatibility**: Natural polymers are generally well tolerated by the human body (i.e., hyaluronic acid has a cell survival rate of 70–80%), but they may cause immunogenic response and microbial contamination.	[92]
		**Biodegradability**: Lack of tunability and an uncontrollable degradation rate. The degradation rate of natural polymers can vary depending on their composition and environmental conditions, i.e., collagen has a degradation rate of about 8 weeks, while alginate has a degradation rate of about 4–6 months.	[120]
		**Mechanical Characteristics**: Weak mechanical strength with respect to the bone load applied. The mechanical stability and elastic modulus of natural polymers can be influenced by their composition and scaffold structure, i.e., collagen has lower mechanical stability compared to other polymers such as PLA and PCL; at the same time, collagen has an elastic modulus of about 0.1–1 GPa, while alginate has an elastic modulus of about 0.01–0.1 GPa.	[121]
		**Other Characteristics**: Difficult to manipulate due to their tendency to swell or break in aqueous solutions. In addition, the formation of a scaffold may require the use of cross-linking agents, such as calcium ions, which can affect the structure and mechanical properties of the polymer, i.e., the formation of a silk scaffold may require the use of cross-linking agents such as ethanol, with a maximum concentration of 70% to prevent material breakdown.	[122]

Scaffolds incorporating MSCs prior to in vivo transplantation encounter specific limitations, necessitating a dual intervention process for MSC harvesting from the iliac crest and subsequent scaffold implantation. Additionally, challenges arise from the potential loss of phenotypic characteristics in the ex vivo culture of MSCs, resulting in diminished osteogenic capacity, and the complexities associated with sterilising cell-seeded scaffolds before implantation [80,81]. Furthermore, in clinical practice, patient variability, including factors such as age, overall health, and genetic background, may significantly influence the scaffold’s efficacy, particularly in terms of its ability to promote osteoblast differentiation [81].

Despite advancements in scaffold design, even the most innovative options, boasting controllable degradation rates, sterilisation feasibility, and the ability to support physiological loads, face significant challenges. Notably, bio-derived silk fibroin scaffolds seeded with MSCs grapple with the inability to control the morphology and pore distribution within the scaffold. The prevalent salt-leaching methods, commonly employed in generating such scaffolds, allow control only over the size of NaCl grains, neglecting their distribution and geometry. Consequently, the resulting scaffolds exhibit random porosity, hindering the uniform distribution of newly formed bone tissue [81].

Therefore, the ideal bone substitute should be both osteoinductive and osteoconductive, exploiting the self-healing capacity of the human bone, while prioritising efficient tissue-engineered solutions. This emerging field focuses on developing regenerative solutions that promote efficient bone healing and prioritise advanced fabrication processes. These processes involve utilising biocompatible materials, such as biodegradable polymers, to construct scaffolds and implants reducing the environmental footprint associated with traditional medical interventions. Furthermore, advanced tissue engineering emphasises optimising fabrication processes, utilising energy-efficient methods like 3D printing, and minimising resource consumption via advanced modelling techniques that prevent extensive experimental campaigns.

Herein, the transformative landscape of personalised bone repair is evolving through an **innovative triad** that harmonises ***bio-inspired design***, ***bio-derived materials***, and ***digital fabrication***. This innovative approach aims to revolutionise traditional methods of addressing bone injuries by seamlessly incorporating principles inspired by nature, advanced digital technologies, and highly compatible materials. Bio-inspired design takes cues from the intricate architectures of natural skeletal structures, envisioning implants that replicate the biomechanical properties of the human body for optimal compatibility. The introduction of digital fabrication technologies, including 3D printing and Computer-aided Design, facilitates the precise and tailor-made construction of scaffolds and implants, ensuring a customised fit aligned with individual patient anatomy. This level of customisation not only guarantees accuracy and snugness but also maximises the mechanical and structural functionality of the implants. The integration of bio-derived materials emphasises a commitment to high-performance practises and introduces an environmentally conscious dimension to the bone repair process. By repurposing bio-derived materials, this triad not only contributes to ecological responsibility but also provides a cost-effective alternative for personalised bone repair solutions.

## 2. Bio-Inspired Scaffold Design

Biomimicry of natural features, which is intended as the ability of the scaffolds to reproduce compositional and structural features of the host tissues, is increasingly considered a guide for the generation of innovative and functional bone substitutes.

A cutting-edge approach has focused recently on the exploitation of biomimicry applied to scaffold architecture design, which aims to design bone substitutes that closely resemble the bone tissue’s anatomical and mechanical characteristics [123].

Indeed, this bio-inspired approach tries to optimise cell-scaffold cross-talks towards the fully functional restoration of bone. As a matter of fact, bio-inspired scaffolds from Nature are considered the future of regenerative therapies for the treatment of bone lesions of critical size, since they target the mimic of the structure/shape (i.e., the bone porosity gradient) and of the mechanical properties of the bone (i.e., ability to bear a physiological load), in addition to mimicking the osteoregenerative capacity of the bone, in order to create more effective bone substitutes than those currently existing [123,124].

The rationale behind the bio-inspired approach is based on the concept that natural systems have already evolved highly efficient and effective solutions for various biological functions. By studying and replicating these natural systems, researchers and engineers could implement novel materials and devices that are optimised for specific applications [125]. In the context of bone regeneration, the bio-inspired approach seeks to mimic the natural structure and function of bone tissue, which has evolved to withstand the mechanical stresses of daily life. By redesigning this structure and function in a synthetic scaffold, researchers aim to implement bone substitutes that can seamlessly integrate with the surrounding tissue and promote the growth of new bone, resulting in more rapid and effective healing [126].

This approach to scaffold design offers several advantages over traditional methods. Bio-inspired scaffolds, by mimicking the natural structure and mechanical properties of bone tissue [127], provide an environment more conducive to bone regeneration, resulting in a more effective and long-lasting bone substitute [128,129].

Moreover, the bio-inspired approach can yield scaffolds that are more biocompatible and less prone to rejection by the body’s immune system, thanks to the use of natural materials already recognised by the body. Additionally, this approach can be cost-effective and eco-friendly, as natural materials are readily available and easily processed into the desired shape and structure [130,131].

### Inspirational Sources

Bio-inspired scaffolds are at the forefront of regenerative medicine for critical-sized bone injuries, representing a promising avenue for the development of bone substitutes that faithfully replicate the geometry and functionality of healthy bone [26]. Researchers have explored a multitude of natural sources for inspiration, with the bone itself being a primary focus. In these studies, efforts have been made to mimic the shape, geometry, size, and distribution of pores found in healthy bone, crucial for the functionality of bone tissue. For instance, healthy human trabecular tissue has ellipsoidal bone gaps with specific dimensions that house a significant number of osteocytes [9,25]. In contrast, osteoporotic bone tissue exhibits altered lacunae morphology and reduced osteocyte quantity, emphasising the importance of accurate replication for functional bone substitutes [132,133,134,135,136,137,138].

The characteristics under consideration extend beyond structural aspects, encompassing chemical and biological features of the bone matrix structure. Researchers aim not only to achieve a porosity consistent with natural bone but also to mimic mineralised collagen fibre bundles for promoting osseointegration and interacting with bone marrow mesenchymal stem cells [139]. While cancellous bone architecture serves as inspiration for scaffolds focusing on mechanical properties, the hierarchical structure of natural bone and its extracellular matrix (ECM) organisation is also emulated for greater bioactivity and to minimise morphological mismatches with the tissue [98,127,139,140,141].

Beyond the animal kingdom plant world and unicellular microorganisms result as alternative sources of inspiration. Initially, plant-based scaffolds are explored to address concerns related to the abundance and biosafety of animal-based resources in conventional bone substitutes. However, challenges arise due to the bio-inert nature of cellulose, hindering predictable bone regeneration [134,142]. Seeking a safer and more predictable regeneration process, researchers drew inspiration from the cross-kingdom adhesion observed in plants and bacteria. Natural microporosity in plants, similar in size and morphology to natural bone, is found to guide the alignment of mammalian cells into bone tissue-like structures, opening new possibilities for reliable bone tissue regeneration [142].

**Figure 4:**
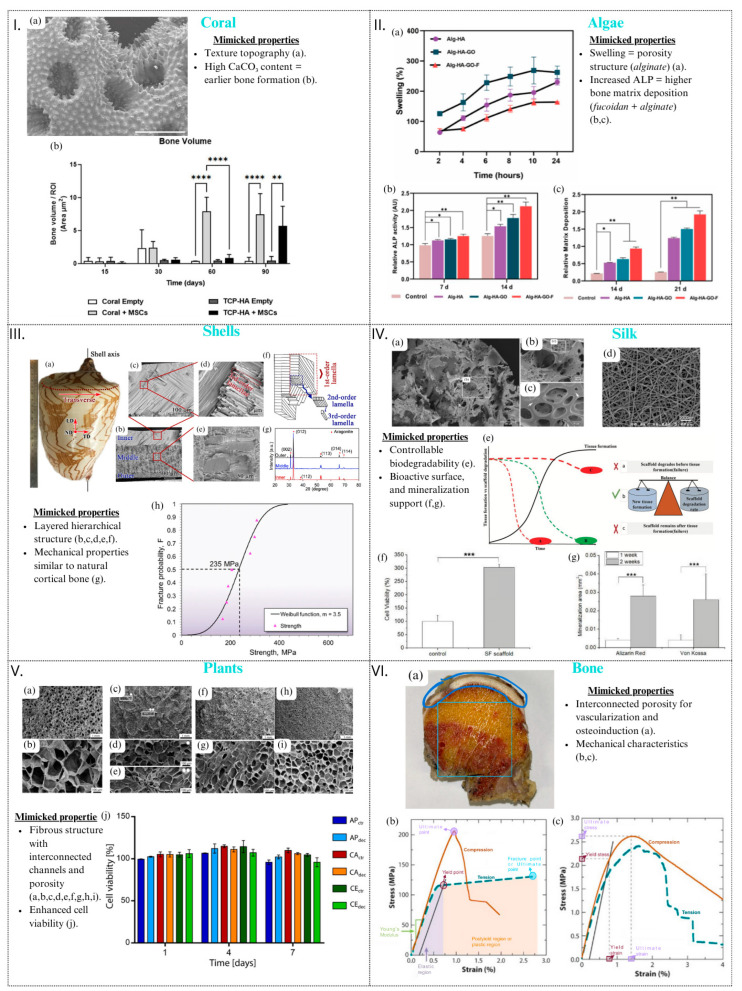
Bio-inspired scaffold concept design (natural source, mimicked characteristics, shapes, and properties). (**I**) Coral scaffolds mimic Pocillopora’s texture and porosity, promoting fibroblastic MSC organisation for early bone formation. (**) *p* < 0.01, (****) *p* < 0.0001, two-way ANOVA. (**II**) Algal scaffolds, combining alginate (Alg) with HA, GO, and fucoidan (F), exhibit swelling properties and ALP activity akin to cortical bone [144]. * *p* < 0.05 and ** *p* < 0.01 compared with the control cells. (**III**) Shell-based scaffolds replicate the hierarchical structure and strength of *C. nobilis* shells, showing multi-scale lamellar features. Overall view of *C.nobilis* shell (**III-a**) and Weibulls statistical plot of compressive strength of *C. nobilis* shell samples (**III-h**). (**IV**) Silk fibroin scaffolds are porous and bioactive, supporting mineralisation and tissue growth. (**IV-d**) FE-SEM images of pure silk nanofibers (6000×; scale bar, 500 nm). *** denotes statistical significance at *p* ≤ 0.001. (**V**) Plant-derived scaffolds, like decellularised apple (AP), carrot (CA), and celery (CE), offer porous structures for enhanced cell viability. (**VI**) Human bone is the key inspiration, mimicking cortical and trabecular features for scaffold strength and porosity. The images (**I**–**III**), (**IV-a**,**IV-b**,**IV-c**), (**IV-e**,**IV-f**,**IV-g**), and (**V**) are adapted, respectively, from [145,146,147,148,149,150] under a CC BY-NC 4.0 license (https://creativecommons.org/licenses/by/4.0/, accessed on 1 August 2024). The image (**IV-d**) is adapted from [151] under a CC BY-NC 3.0 license (http://creativecommons.org/licenses/by-nc/3.0/, accessed on 3 August 2024). Layouts have been modified from the original.

A perspective of the main natural sources of bio-inspiration for bone scaffolds and their mimicked characteristics, shapes, and properties (mechanical, structural, biological, and chemical) is shown in Figure 4. This figure highlights how natural sources of inspiration, including corals (Figure 4I), algae (Figure 4II), shells (Figure 4III), silk (Figure 4IV), plants (Figure 4V) [143], and human bone itself (Figure 4VI), are employed not only as innovative materials for the fabrication of scaffolds in bone regeneration but also as models for their intrinsic characteristics—such as structural organisation, composition, mechanical properties, biodegradability, and porosity—which are leveraged to design scaffolds with optimal properties for bone regeneration applications.

## 3. Bio-Derived Materials

### Advances in Bio-Inspired Material Choice

The utilisation of bio-derived and biomimetic materials for bone scaffolds represents innovative and eco-friendly approaches in regenerative medicine, also contributing to enhanced integration with the host tissue, promoting cellular interactions, and facilitating the regeneration of bone [152].

Herein, the selection of hydroxyapatite, collagen, and alginate as materials for scaffold generation is based on their ability to mimic the structural and functional properties of natural bone tissue. Hydroxyapatite is a mineral component of bone, while collagen and alginate are two types of proteins that are also found in bone tissue. The combination of these materials could be exploited to generate scaffolds with bone-like functions that closely emulate the structure and properties of natural bone tissue.

These materials offer a high degree of versatility, which enables researchers to generate scaffolds with a variety of bone-like functions. For example, scaffolds made from these materials can be designed to facilitate the growth of new bone tissue, provide support for weakened or damaged bones, and even deliver drugs or other therapeutic agents to the site of bone injury or disease.

**Figure 5:**
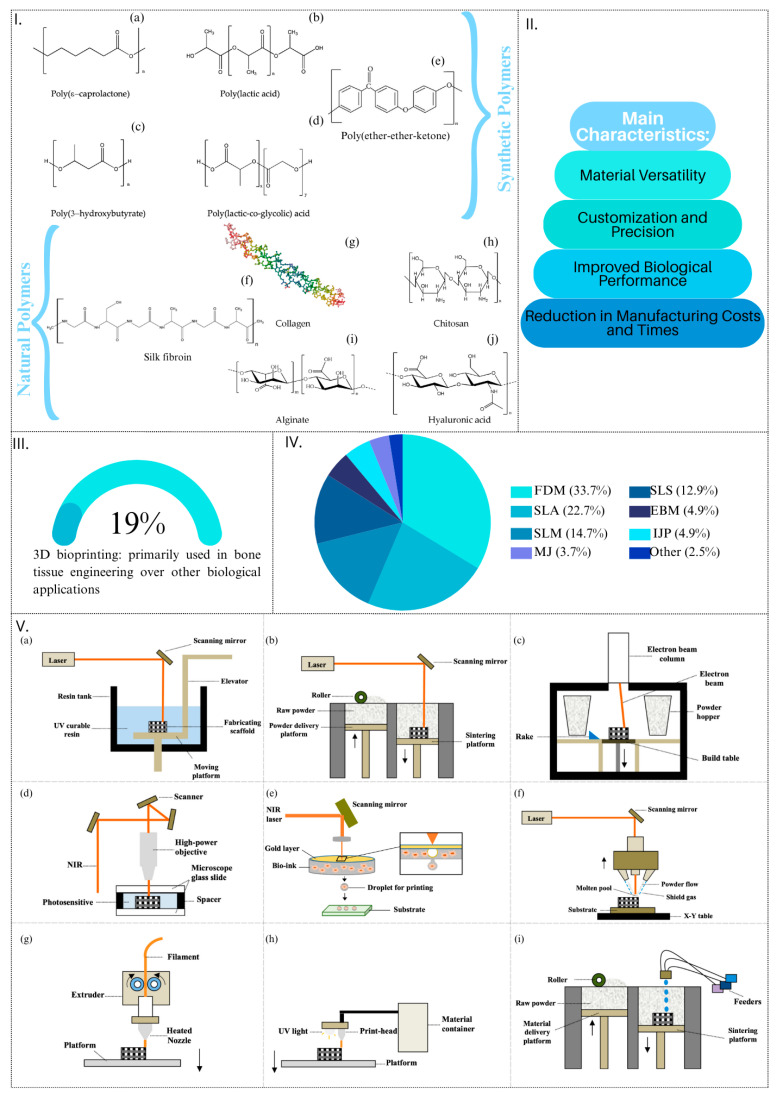
Advanced 3D-printing techniques for bone scaffolds. (**I**) Synthetic (PCL, PLA, PHB, PLGA and PEEK, respectively **I-a**, **I-b**, **I-c**, **I-d** and **I-e**) and natural (silk fibroin, collagen, chitosan, alginate and hyaluronic acid, respectively **I-f**, **I-g**, **I-h**, **I-i** and **I-j**) polymeric materials commonly used for bone scaffold fabrication [94]. (**II**) Main advantages of 3D-printing techniques for bone scaffolds compared to conventional techniques. (**III**) Percentage of 3D bioprinting used in bone tissue engineering applications [162]. (**IV**) Percentages of various 3D-printing methods studied for bone scaffolds: SLA (stereolithography), SLM (Selective Laser Melting), SLS (Selective Laser Sintering), FDM (Fused Deposition Modelling), EBM (Electron Beam Melting), MJ (Material Jetting), IJP (InkJet Printing), and Other (2PP (two-photon polymerisation) and LENS (laser-engineered net shaping)) [162]. (**V**) Innovative 3D-printing techniques for bone scaffold manufacturing: laser-based technologies (SLA (**V-a**), SLS/SLM (**V-b**), EBM (**V-c**), 2PP (**V-d**), laser-based bioprinting (**V-e**), and LENS (**V-f**)) and extrusion-based technologies (FDM (**V-g**), MJ (**V-h**), and IJP (**V-i**)). The images (**I**) and (**V**) are adapted respectively from [94,162], under a CC BY-NC 4.0 license (https://creativecommons.org/licenses/by/4.0/, accessed on 13 August 2024). Layouts have been modified from the original.

Among the array of promising bio-derived and bio-waste-derived materials, polymers stand out as pivotal contributors in the generation of innovative scaffolds for bone substitutes. They fall into two broad categories: synthetic polymers, including polylactic acid (PLA), poly lactide-co-glycolide (PLGA), and polycaprolactone (PCL), and natural polymers, such as collagen, silk fibroin, and alginate (Figure 5I).

As pertains **synthetic bio-derived polymers**, they exhibit favourability due to their remarkable versatility in both chemical and physical properties, offering solutions to challenges associated with alternative materials in bone substitute fabrication [153]. For instance, traditional choices like metals, including titanium alloys [133,136], have been employed in synthetic bone substitutes, but their scaffolds exhibit a high Young’s modulus (about 100 GPa), leading to stress shielding and subsequent bone resorption unless porosity control methods are applied [136]. Conversely, highly promising synthetic polymers for crafting bio-inspired scaffolds include polylactic acid (***PLA***), derived from renewable resources such as corn starch or sugarcane, [140,154] and poly lactide-co-glycolide (***PLGA***) combined with Gly-Phe-Hyp-Gly-Arg (GFOGER) [155]. These polymers facilitate the imitation of the bone’s extracellular matrix, effectively reducing morphological mismatches between synthetic bone substitutes and bone tissue. The incorporation of GFOGER with these polymers not only aids in mimicking the natural bone hierarchical structure but also enhances overall bioactivity [133].

A notable synthetic polymer gaining widespread use in biomimetic bone scaffold generation is polycaprolactone (***PCL***), produced from the fermentation of renewable feedstocks such as certain types of bacteria or fungi, and polylimonene carbonate (***PLC***), derived from limonene, a compound found in citrus fruit. Often utilised alongside additional therapeutic agents like Metallic Ions as Therapeutic Agents (MITAs) [156,157], PCL brings about biological advantages and potential enhancements in mechanical and antimicrobial properties. The most frequently used metallic ions in conjunction with PCL include strontium (Sr), magnesium (Mg), and zinc (Zn). Research demonstrates that Sr and Mg, within specific limits, promote osteogenic differentiation of MSCs and inhibit osteoclastic activity. Zinc contributes excellent swelling properties to the scaffold, facilitating cellular growth without compromising mechanical properties. Moreover, magnesium imparts superior compressive strength to the polymeric scaffold, aligning with natural bone properties, while zinc influences compressive resistance, elasticity, and stiffness, effectively increasing tensile Young’s modulus [156,157].

On the alternative front, various **natural bio-derived polymers**, including silk fibroin, collagen, alginate, chitosan, and gelatin, have proven instrumental in forging innovative bio-inspired bone substitutes. ***Silk fibroin*** stands out as a polymer of choice for crafting cutting-edge scaffolds, boasting immunocompatibility, cytocompatibility, high mechanical strength, thermal stability, and facile chemical modification. Silk fibroin, with a tensile strength of around 300–740 MPa, offers better mechanical resistance than PLA, which has a tensile strength of around 50–70 MPa. This makes silk fibroin more suitable for load-bearing applications. In tandem, ***collagen***, a key component of natural bone, complements silk fibroin by contributing to controlled biodegradation, haemostatic properties, biocompatibility, and the ability to promote cell adhesion. Combining these two polymers yields a flexible composite scaffold, especially potent when synergised with bioactive glasses (BAGs). The integration of BAGs enhances bone formation through chemical interactions with the surrounding bone tissue. The composition of BAGs, primarily comprising CaO-SiO_2_, endows them with outstanding osteoconductive and osteoinductive properties. Osteoconductive materials provide a scaffold for bone growth, while osteoinductive materials actively stimulate stem cells to differentiate into bone-forming cells, promoting new bone formation. While BAGs inherently exhibit stiffness and fragility, their incorporation into the composite scaffold alongside collagen and silk fibroin serves to emulate the natural structure of the bone matrix effectively [158].

These materials emerge as a milestone in the realm of bone bio-inspiration, finding application in the generation of in vitro bio-inspired bone models. These material models meticulously replicate the composition and arrangement of the bone extracellular matrix, addressing a broader objective within bone bio-inspiration. This objective strives to develop innovative substitutes and models facilitating the study of physiological bone remodelling while adhering to the principles of replacement, reduction, and refinement (3R). This holistic approach aims to ultimately curtail the need for animal experiments, thanks to the advancements. Moreover, this bio-inspired microstructure, modelled after natural bone, features optimal porosity to facilitate endothelial cell migration and blood vessel formation, alongside promoting bone regeneration. Certain bio-inspired materials are designed to release vascular endothelial growth factors (VEGFs) in a controlled manner, thereby stimulating angiogenesis. This controlled release enhances vascularisation at the site of bone regeneration, supporting the healing process and improving overall regenerative outcomes [159,160].

Within the realm of synthetic bio-based polymers (i.e., PCL, PLA, and PLGA), ***hydroxyapatite***, both synthetised and found in natural bone, is often used as a coating to provide a mineral component that enhances the biocompatibility of scaffolds.

In contrast, natural bio-derived polymers, such as ***chitosan*** (derived from chitin, which is found in the exoskeletons of crustaceans) and ***gelatin*** (derived from collagen) base scaffolds, are less exploited due to the lack of biological activity, even if they display appropriate biocompatibility and mechanical properties.

Referring to **bio-waste-derived materials** for bone scaffold implementation, the main sources are the agricultural or industrial processes. Indeed, ***lignin*** particles from wood processing waste have been recently explored as reinforcing material, given lignin’s intrinsic mechanical resistance [161]. Additionally, waste from fish processing, like ***fish scales***, has been investigated for its calcium content, making it a valuable candidate for bone scaffold development. ***Eggshell-derived calcium carbonate***, sourced from waste eggshells, presents an opportunity to utilise the rich calcium resources for scaffold generation. ***Bamboo*** fibres and particles, considered waste in certain processes, have been explored for their potential contribution to composite scaffolds. Furthermore, ***waste from biogas*** production, such as digestate, containing organic and inorganic components, holds promise as a resource for scaffold development.

The mentioned biomimetic/bioderived scaffolds, while offering significant advantages, present ***potential risks and failure modes*** that must be carefully managed. The exploitation of natural sources significantly reduces the risk of immune reactions, improving the inflammatory/rejection risk; however, scaffold degradation inconsistencies, such as uneven biodegradation rates, pose a challenge. If the scaffold degrades too quickly, it may fail to provide adequate support for tissue regeneration; if it degrades too slowly, it may impede new tissue formation or result in prolonged inflammation. Mechanical failures under physiological conditions, such as insufficient tensile strength or fatigue resistance, can lead to scaffold collapse or deformation, especially in load-bearing applications. These risks highlight the need for thorough in vitro and in vivo testing to assess biocompatibility, mechanical performance, and degradation behaviour under realistic biological conditions.

## 4. Digital Fabrication

The utilisation of natural and recycled materials for bone scaffolds is seamlessly complemented by digital fabrication technologies, introducing a sophisticated dimension to innovative scaffold development. Digital fabrication methods, including 3D printing and Computer-aided Design (CAD), serve as instrumental tools in the precise conversion of these bio-derived and bio-waste materials into intricately designed and customizable scaffolds. The amalgamation of bio-waste utilisation and digital fabrication delineates a synergistic trajectory toward environmentally conscientious and technologically advanced bone scaffold development.

### 4.1. Advances in Digital Design of Architected Shapes

As discussed in Section 2, the intricate architectural shape of bone forms the basis for the development of functional and innovative bio-inspired scaffolds. This architectural complexity, spanning from the nano-scale to the macro-scale, is intricately linked to the mechanical properties of the entire skeletal structure [132]. The ***hierarchical architecture*** of bone plays a pivotal role in activating mechano-transduction phenomena at the cellular level, facilitating bone adaptation to external loads, and enabling self-regeneration and repair in the face of limited damage. To be efficacious in mending bone fractures, bio-inspired bone substitutes must exhibit a meticulously controlled hierarchical structure aligned with the precise mechanical properties of natural bone tissue [136,153,163]. For instance, studies report that scaffolds with porosities of 70–90% lead to superior bone ingrowth and osseointegration, supporting mechanical stability in vivo [27,28,164].

A critical property that scaffolds must possess to ensure the development of new bone and a robust bone–implant interface is the emulation of bone mineral composition, encompassing the morphology and distribution of scaffold ***multi-scale pores***. Scaffold porosity, particularly in the range of 300–700 micrometres, has been shown to significantly enhance osteoconduction and neo-angiogenesis, as evidenced by increased bone mineral density in in vivo models [53]. Numerous studies underscore the positive impact of highly porous structures, enhancing bone penetration, optimal osseointegration, and overall biomechanical performance of the bone substitute [165]. The choice of an ordered porosity, resembling a channel, enhances cell seeding efficiency, promotes viable cell distribution within the scaffolds, and facilitates nutrient transport. Pore interconnectivity, ideally above 70%, further facilitates cell migration and nutrient diffusion, leading to improved osteogenic outcomes in scaffolds tested in pre-clinical trials [164]. Ordered porosity often supports the regrowth of osteonic structures, while randomly oriented porosity is more prone to favour the formation of new bone tissue [166,167]. In addition to overall porosity, the shape, interconnection, and arrangement of pores are pivotal factors, influencing osteoconductive processes and facilitating optimal cell migration [155,163,168]. Some research endeavours focus on replicating bone porosity, exploring the utilisation of a decellularised plant (cellulose) tissue scaffold for bone tissue regeneration [161]. This scaffold boasts a hierarchical porous structure with pore sizes ranging from a few micrometres to several hundred micrometres, optimised to support nutrient and waste transport, as well as cell infiltration and proliferation [127]. Fabricated by preserving the extracellular matrix (ECM) architecture of the plant tissue, the scaffold guides the alignment of human MSCs to reform bone tissue. Data from in vivo studies demonstrate that scaffolds with hierarchical porosity significantly improve bone matrix formation compared to non-hierarchical designs [169,170]. While the scaffold alone is insufficient for producing healthy and functional bone tissue, its bioactivity is enhanced by using nano-amyloids to promote cell adhesion, vitality, and proliferation. Additionally, nano-hydroxyapatite crystals deposited on the amyloid further stimulate the osteogenic differentiation of pre-osteoblasts. Studies report a threefold increase in osteoblast differentiation on amyloid-coated scaffolds compared to uncoated controls [171]. This hierarchical design leverages the natural intrinsic microporosity of the plant scaffold, the dedicated microstructure of natural plants, and the high bioactivity of nano-amyloid/hydroxyapatite coatings to successfully regenerate trabeculae within the scaffold [142]. In the realm of natural-type matrices, silk fibroin emerges as a versatile material with the capacity to adopt various non-fibrous forms, including hydrogels, films, microspheres, and scaffolds. These diverse forms can mimic the architecture of spongy bone-like or cortical porous structures, akin to type I collagen, demonstrating the ability to support the adhesion and proliferation of mesenchymal stem cells (MSCs) [132,172]. Through the combination of silk fibroin with collagen and processing it via an Espin Nano (V2HC2) electrospinning device, crystalline nanofibres containing bioactive glass particles can be created. The resultant multifunctional bone fibres exhibit a stratified structure closely resembling the sequence of neo-bone tissue and apatite formation. This structure is stabilised by the chemical interaction between collagen and silk fibroin through amino groups, as well as the presence of CaO-SiO_2_ particles. Overall, the utilisation of silk fibroin, collagen, and bioactive glass particles in conjunction with the Espin Nano (V2HC2) electrospinning device holds promise as an effective approach for developing bone tissue engineering scaffolds that faithfully mimic the intricate structure of natural bone tissue [158].

In contrast to drawing inspiration solely from bone structure for designing hierarchical scaffolds that facilitate bone tissue regeneration, researchers have explored alternative methodologies to stimulate the growth of new bone tissue. One such alternative involves the utilisation of ***triply periodic minimal surfaces***, such as the gyroid, as a potential scaffolding for bone tissue. The gyroid, a triply periodic minimal surface found in various natural materials, including butterfly wings, sea sponges, and bone, provides an environment that promotes the growth of new bone tissue and facilitates the transfer of loads and stresses akin to natural bone. This unique structure helps mitigate stress shielding, a phenomenon observed when the Young’s modulus of the implant exceeds the elastic modulus of the bone. Additionally, studies indicate that the hierarchical structure of the gyroid, when filled with PLA, yields bone density distributions and properties comparable to those of natural bone [154]. For instance, gyroid-PLA scaffolds showed porosity in the range of 70–75%, similar to cancellous bone, allowing for improved bone regeneration [173]. This innovative approach, seamlessly integrating digital fabrication techniques, showcases a forward-thinking paradigm in bone scaffold development, ensuring precision and efficacy in replicating intricate biological structures. Simultaneously, the GrasshopperTM software serves as a tool for the arbitrary generation of space, manifested as interconnected cylinders whose shapes are subsequently rounded and smoothed using the CocoonTM software. This process yields scaffolds with varying trabecular thicknesses and porosity, with cylindrical geometry deemed most appropriate for the study. Long bones, often prone to fractures, inherently possess a cylindrical geometry, characterised by a dense external microstructure (cortical bone) and a porous internal microstructure (trabecular bone) [138].

Exploring the cutting edge of bone regeneration research, the current forefront involves the development of ***bio-inspired scaffolds derived from machine learning***. These anisotropic porous bone scaffolds leverage a self-learning convolutional neural network model. Anisotropy is crucial here, mirroring the irregular shapes and mechanical anisotropy inherent in native bone structures across different scales, adding complexity to scaffold design for optimal bone regeneration [174]. Researchers initially increase scaffold anisotropy by adjusting the geometrical parameters of the gyroid cellular structure [154]. However, the limited number of design variables in gyroid scaffolds, and triply periodic minimal surface (TPMS) scaffolds in general [175], restrict the design space for anisotropic mechanical properties. To overcome this limitation, advanced mathematical algorithms like the Voronoi algorithm have been employed to design irregular and non-periodic bone scaffolds, broadening the design space but complicating the algorithm [176]. The use of Voronoi algorithms also enables the development of a novel gradient anisotropic design method for Voronoi porous structures, which exhibits adaptable profiles, controllable gradients, and freely orientable anisotropic elastic behaviours aligned with the stress field [177]. In recent decades, there has been a surge in employing neural network techniques for the digital design of scaffolds with mechanical properties akin to native bone [9]. While still under study, these techniques show great potential by significantly increasing the number of design variables, which is crucial for porous scaffold design. Unlike conventional frameworks with four independent design variables corresponding to scaffold thicknesses, machine learning enables the use of 3^4^ possible design variables for the optimisation of anisotropic bone scaffolds (with three potential values for each of the four thicknesses). To address the resulting increase in calculation time, a novel self-learning convolutional neural network (CNN)-based optimisation framework has been developed, enhancing calculation efficiency [176]. The use of CNN in scaffold design significantly improves the accuracy in mimicking natural bone’s mechanical properties, especially anisotropy, which is crucial for successful bone regeneration [176].

### 4.2. Advances in Digital Fabrication Techniques

The synergy between digital design and digital fabrication is pivotal in realising the full potential of innovative bio-derived bio-inspired scaffold structures for bone regeneration.

Indeed, innovative bio-inspiration strategies have made it necessary to use new manufacturing techniques, which can introduce porosity gradients and structures that mimic bone architecture.

In recent decades, various traditional techniques have been employed in the manufacturing of bio-inspired bone substitutes designed for the repair of bone lesions, including critical-sized injuries. For instance, promising silk fibroin scaffolds seeded with mesenchymal stem cells have demonstrated potential for facilitating the differentiation of MSCs into bone tissue cells like osteoblasts and osteocytes. However, limitations arise in the production of silk fibroin scaffolds using the salt-leaching method, which, while achieving the desired porosity comparable to healthy bone (250–300 µm), lacks control over pore distribution throughout the scaffold volume [53,153].

This deficiency in porosity control impedes the uniform arrangement of newly formed bone tissue across the entire implant volume. To overcome this challenge, the most effective technology to date for implementing scaffolds with controlled and homogeneous porosity, along with precise mechanical properties at the microstructural level, is the utilisation of advanced 3D-printing and 3D-bioprinting technologies. Leveraging additive manufacturing principles, 3D printing enables the layer-by-layer construction of objects directly from a 3D CAD (Computer-aided Design) model [138]. The digital fabrication and scaffold modelling process begins with CAD (Computer-aided Design) modelling, where the scaffold’s geometry is designed to replicate the natural architecture of bone tissue, including key features such as porosity and mechanical properties. The 3D printing setup utilises additive manufacturing techniques, such as Fused Deposition Modelling (FDM) or stereolithography (SLA), allowing precise control over the scaffold’s external shape and internal structure. During fabrication, pore size and distribution are optimised to promote nutrient flow and cell infiltration, with layer-by-layer construction ensuring structural integrity and uniformity. This workflow ensures the realisation of scaffolds tailored to specific biological and mechanical requirements.

Notably, 3D-printing and 3D-bioprinting techniques have gained widespread adoption for scaffolds in polymeric materials [153,174], demonstrating their efficacy in producing structures that emulate the hierarchical nature of bone tissue. One of the major advantages of 3D printing is the **precise control over external shape and internal architecture**, which extends to critical characteristics such as pore shape, interconnection, permeability, and stiffness [178]. This level of precision is difficult to achieve with conventional techniques like foaming, sacrificial templating, negative templating, and slip casting.

In addition to architectural precision, 3D printing offers significant benefits in terms of **material versatility**. It supports the use of a wide range of biomaterials, especially different types of polymers. This flexibility enables the design of scaffolds tailored to meet the specific mechanical and biological requirements of different bone tissue environments. Moreover, 3D printing allows for customisation and precision, permitting the creation of patient-specific scaffolds based on medical imaging data, ensuring a precise fit and optimised functionality for individual bone defects [179].

Another key advantage is the **improved biological performance** offered by 3D printing. The technology enables precise control over pore architecture, which enhances cell attachment, proliferation, and nutrient diffusion, ultimately promoting more effective tissue integration and regeneration [179]. Additionally, 3D printing results in **economic advantages**, as it minimises material waste and reduces production times, making the process more cost-effective.

Taken together, these advantages mark a significant stride toward personalised and effective bone tissue engineering (Figure 5II).

Herein, extensive research has been conducted on 3D-printed scaffolds employing various materials, with PCL scaffolds emerging as effective solutions for healing bone lesions in skeletal areas not subjected to high loads, given the material’s limited stiffness and strength [133,180].

The techniques employed in 3D printing, particularly 3D bioprinting, are widely used for the creation of bio-inspired bone scaffolds, more so than in other biological applications (Figure 5III). Among the 3D-printing techniques for bone scaffold fabrication, Fused Deposit Modelling (FDM) is the most commonly used [181], followed by stereolithography (SLA) and Selective Laser Melting (SLM) (Figure 5I,V). However, the application of all 3D-printing techniques varies depending on the type of polymer material used, as illustrated in Figure 5V [178].

Extrusion-based methods, such as direct inkjet, bio-ink, and electrospinning printing, are commonly utilised for polymer scaffolds like PCL and PLA [53]. These techniques involve the layer-by-layer deposition of wires or rods in a specific order. However, these extrusion-based methods face limitations related to the shape and diameeer of the nozzle and its efficiency in material extrusion. Consequently, alternative techniques, including those based on photopolymerisation such as stereolithography, digital light processing, and two-photon polymerisation, have been explored. These methods enable sequential cross-linking of a light-curing polymer using a light source, allowing for the creation of bio-inspired scaffolds with patient-specific shapes. It is important to note that these photopolymerisation techniques are restricted to materials obtained through the customisation and processing of photosensitive polymers.

Additionally, techniques like selective sintering, melting, or fusion, which utilise energy sources such as ion beams, lasers, electron beams, or thermal sintering, provide high control over the scaffold’s final shape and are applicable to polymers. However, the high cost associated with these energy sources limits their widespread use. To address this limitation, a technique known as binder jet has been recently employed, replacing the high-energy source with a liquid binder like water or phosphoric acid [133]. Table 2 offers a comparative analysis highlighting the advantages and limitations associated with distinct 3D-printing methodologies utilised in the implementation of biomimetic bone scaffolds.

Despite the advantages of 3D printing, achieving uniform porosity at a macroscopic scale remains challenging due to the inherent limitations of both the printing process and material properties. In extrusion-based techniques, factors such as nozzle shape and size, material flow variability, printing speed, layer thickness, and temperature can lead to irregular pore sizes and shapes, particularly at the microscale [182,183,184]. These inconsistencies affect the scaffold’s mechanical properties and its biological performance. Ensuring an optimal pore size that mimics the natural hierarchical structure of bone is essential for facilitating nutrient transport, cell infiltration, and bone regeneration [189].

Additionally, the choice of material for 3D printing often restricts the design and functionality of bio-inspired scaffolds. Many polymers may fail to adequately replicate the mechanical properties of natural bone or may degrade at rates that are either too fast or too slow, thereby compromising scaffold integration with host tissue. Incorporating bioactive agents to enhance scaffold properties poses further challenges, as achieving a homogeneous distribution of these agents during the 3D printing process can be difficult [190].

## 5. Advanced Numerical Modelling for a Novel Triad of Bio-Inspiration, Digitalisation, and Renewability in Bone Repair

The integration of advanced numerical modelling stands at the forefront of a ground-breaking triad, seamlessly merging principles of bio-inspiration, digitalisation, and environmental responsibility in the realm of bone repair. This novel approach leverages sophisticated computational techniques to simulate, analyse, and optimise the intricate processes involved in personalised bone regeneration.

### 5.1. Numerical Modelling of Bone–Scaffold Interaction

Within the realm of computational modelling, Finite Element (FE) Analyses and the progressive integration of artificial intelligence (AI), encompassing machine learning (ML)-based techniques, stand as prominent methodologies. Computational modelling provides a pivotal advantage in its capability to simulate and comprehend new bone formation within scaffold tissue engineering, not yet feasible in in vivo studies, where random factors, such as the distribution of pores within the scaffold and the material composition, introduce complexities [191].

The adoption of numerical modelling for the investigation of intricate bio-inspired scaffolds necessitates a ***multi-scale perspective*** [26]. The integration of machine learning and multi-scale modelling occurs at two levels: the parameter level and the system level. At the parameter level, this integration involves constraining design spaces, identifying critical values, and analysing their sensitivity. At the system level, it leverages the underlying physics to explore system dynamics and constrain design parameters. Machine learning equips researchers with tools for enhancing training data, mitigating overfitting, managing ill-posed problems, creating surrogate models, and quantifying uncertainty. Meanwhile, multi-scale modelling synthesises the underlying physics to identify relevant features, explore their interactions, elucidate mechanisms, bridge different scales, and understand the emergence of functional properties [192]. This approach involves the consideration of structural organisation across multiple levels, spanning from the macroscopic to the nanoscopic scale [193]. The inherent architecture of bio-inspired constructs is optimally understood through multi-scale modelling, enabling the exploration of interactions among scaffold components, including cells, extracellular matrix, and the scaffold itself [194]. This holistic understanding offers insights into enhancing the scaffold’s overall functionality for specific biomedical applications [193,195]. Furthermore, multi-scale modelling plays a crucial role in pinpointing critical parameters influencing scaffold performance. The analysis of the scaffold at different scales aids in determining the most significant parameters for achieving desired properties, such as mechanical strength, biocompatibility, and controlled release of bioactive molecules [193,196].

Nevertheless, it is imperative to acknowledge that many existing numerical models entail a substantial computational cost, particularly when addressing multi-scale challenges associated with modelling bone substitutes, especially for extensive fractures. Conventional FE analyses, while powerful, come with limitations that significantly escalate computation [176]. One such limitation is the need to solve both macroscopic and microscopic governing equations concurrently to ascertain strains at both scales. Additionally, the dependence of bone remodelling results within each representative volume element (RVE) on microscopic strain energy density (SED) [197], which is contingent on the macroscopic strain, further contributes to the computational demands. The repeated execution of FE analyses across all RVEs amplifies the overall computational burden.

Hence, a machine learning (ML)-based approach has been proposed for predicting bone ingrowth outcomes in bulk tissue scaffolds. This methodology has proven to be a highly effective tool not only for anticipating in vivo bone tissue regeneration in subject-specific scaffolding systems but also for efficiently monitoring bone substitutes seeded with mesenchymal stem cells, navigating the intricacies of seeding, differentiation, and proliferation at both macroscopic and microscopic levels [176,198]. The combination of FE and ML methods is used to predict structures of architecture interlocking [199].

To validate the precision and efficacy of the ML-based time-dependent prediction of the bone ingrowth model, a comparative analysis with the conventional multilevel FE model has been conducted [198]. Recent research underscores the superior accuracy and computational efficiency of machine learning (ML) techniques compared to traditional FE procedures. In predicting material mechanical properties, ML models achieved an accuracy of 98.5%, outperforming FE procedures, which achieved an accuracy of 94.5%. Furthermore, the computational cost of the ML approach is 3–4 orders of magnitude lower than the FE approach. Neural networks (NNs) trained on a suitable database enable high accuracy while significantly reducing computational costs for each operation. For instance, in one study, the micro-FE analysis sampling needed for training three NNs could be executed in parallel, a capability not feasible with conventional methods that rely on sequential micro-FE analyses within a designated time period [176].

Once appropriately trained with high accuracy, neural networks become invaluable for inverse identification, demonstrating computational efficiency superior to conventional FE techniques [176,198]. However, despite the demonstrated effectiveness and efficiency of the ML-based model in predicting bone remodelling within scaffolds, certain limitations exist. Firstly, the proposed ML-based model utilises Wolff’s law at the microscopic level to assess the appropriate bone density change in the scaffolds, focusing on bone remodelling after a healing process (3 months) where mechanical stimulus plays a crucial role in the bone formation process. Additionally, assumptions include uniform MSC seeding into scaffold pores and the uniform formation of soft tissue on the porous scaffold surface. It should be noted that alternative ML-based methods can be implemented. Secondly, scaffold degradation is not considered, although this can be mitigated by selecting a material with controlled and slower degradation than the new bone tissue formation time, such as a silk fibroin scaffold [198]. Finally, ML techniques are inherently contingent on the quality of input data, including the accuracy of manual segmentation. Precise and complete segmentation is crucial for optimal ML model performance, emphasising the necessity of automated segmentation techniques [176].

### 5.2. Advances in Validation Strategies

**Figure 6:**
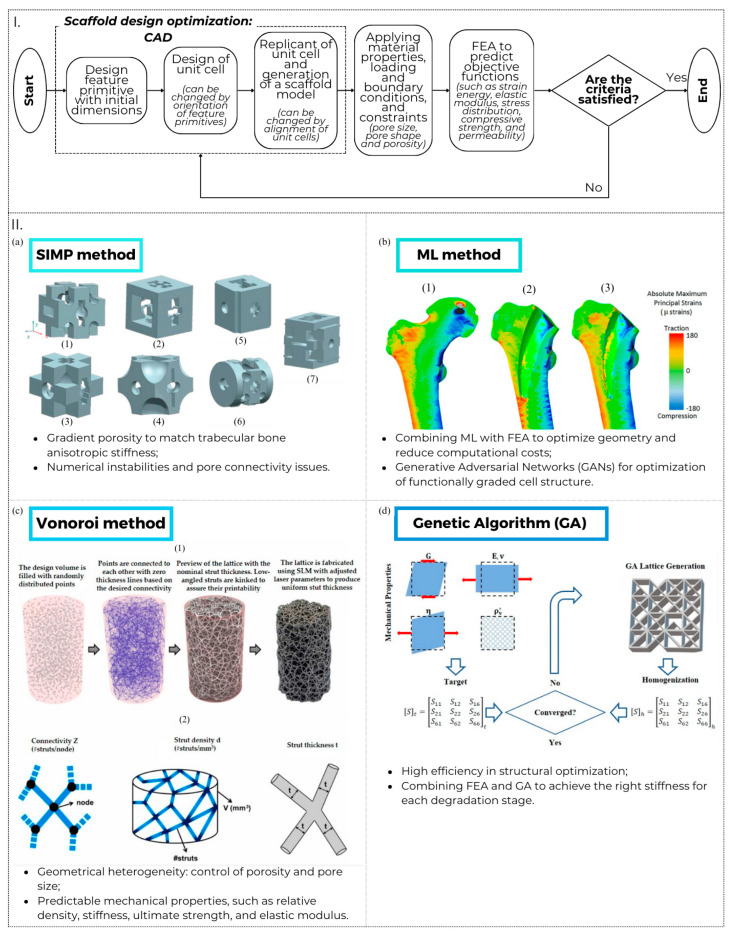
Advanced Bio-inspired Scaffold Modelling Strategies Overview. (**I**) Optimisation process of bone scaffolds (with attention to internal porous architecture). FEA (Finite Element Analysis) and CAD (Computer-aided Design) [162]. (**II**) Current methods in the optimisation of bone scaffolds. (**II-a**) The SIMP method designs complex 3D structures with gradient porosity to optimise stiffness, but faces issues like numerical instability and poor pore connectivity, with optimised cell models (a.1–a.7) shown in different positions. (**II-b**) ML models predict material properties and optimise scaffold geometry, comparing strain distribution in bone models: intact (b.1), with the original stem (b.2), and the new design (b.3). (**II-c**) VTM models irregular, customizable lattices mimicking bone tissue, showing the design of lattice structures (c.1,c.2). (**II-d**) GA optimise scaffold design by selecting parameters for desired stiffness, showing the iterative homogenisation process that optimises material properties (*G*,*E*,*ν*,*η*) [202,203,204,205,206,207,208,209,210,211,212,213,214,215,216,217,218,219,220]. The elements (**a**–**d**) of image (**II**) are adapted from [202], under a CC BY-NC 4.0 license (https://creativecommons.org/licenses/by/4.0/, accessed on 24 July 2024). Layouts have been modified from the original.

While the versatility of advanced modelling allows for capturing the complex interactions between the scaffold, cells, and extracellular matrix, it is crucial to emphasise the importance of proper validation strategies to ensure the reliability and accuracy of these models (Figure 6). Validating the computational predictions against experimental data and in vivo studies is paramount for establishing the credibility of the modelling outcomes [200].

The bio-derived nature of these scaffolds introduces additional complexities, such as variable degradation rates and intricate compositional nuances, which further underscore the need for robust validation methodologies. Integrating experimental data derived from bio-derived scaffold prototypes into the modelling process enhances the predictive capabilities and ensures that the designed scaffolds align with the desired biological and mechanical outcomes.

Advanced modelling strategies have emerged as essential tools for optimising the design and performance of bio-inspired bone scaffolds. These strategies enable precise control over scaffold parameters such as internal porous architecture (Figure 6I), mechanical properties, and biological functionality. **Finite Element Analysis (FEA)** and **Computer-aided Design (CAD)** have become central to scaffold optimisation processes, allowing for the simulation of mechanical behaviour and prediction of scaffold performance under physiological conditions [10,162,200].

Several modern methodologies are employed in scaffold optimisation, each with distinct advantages and limitations. The **Solid Isotropic Material with Penalisation (SIMP) method** allows for the creation of complex multi-scale structures with gradient porosity, which is critical for matching the anisotropic stiffness of human trabecular bone [201]. However, the SIMP method can suffer from numerical instabilities, such as tessellation issues and grid dependence, which necessitate additional constraints to ensure pore connectivity. Despite these challenges, SIMP remains a valuable approach for designing scaffolds that mimic the intricate architecture of bone (Figure 6II-a).

Machine learning (ML) techniques have also been incorporated into scaffold design, enabling the identification of patterns in complex datasets such as medical images. By combining **ML** with **FEA**, it is possible to optimise scaffold geometry and reduce computational costs. Additionally, **Generative Adversarial Networks (GANs)**, when applied to **Inverse Homogenisation (IH)** mapping, can optimise functionally graded cell structures, improving intelligent additive manufacturing processes (Figure 6II-b).

The **Voronoi Tessellation Method (VTM)** has become another important tool for modelling irregular, open-hole structures that mimic bone properties. VTM provides geometric heterogeneity, which is essential for creating biomimetic shapes by controlling scaffold porosity and pore size. This technique allows for the generation of customisable lattice structures that predict mechanical properties such as stiffness, ultimate strength, and elastic modulus, further advancing scaffold design (Figure 6II-c).

Finally, **Genetic Algorithms (GAs)** offer high efficiency in structural optimisation by combining FEA with multi-objective optimisation approaches. GAs are frequently used to select key design parameters, such as fibre diametre and spacing, that impact scaffold stiffness during various stages of degradation. Moreover, they are instrumental in solving nonlinear optimisation problems, enabling scaffold designs to meet both mechanical and biological requirements. For instance, GAs facilitate scaffold optimisation for primary stability and can generate automated solutions for preoperative planning, making them indispensable in scaffold design workflows (Figure 6II-d).

As the field continues to evolve, ongoing efforts to refine and validate these advanced modelling strategies will be instrumental in translating theoretical insights into practical applications.

Firstly, for the ***validation of bone shape recovery***, numerical methods rely significantly on insights derived from in vivo studies of bio-inspired scaffolds. The meticulous control of porosity parameters, including average pore size, distribution, type (close or open), and interconnection, holds paramount importance in determining the efficacy of synthetic bone substitutes; these porosity characteristics are intricately linked to the cross-linking composition and density of the construct. Electron microscopy techniques, such as SEM and TEM, are conventionally employed to obtain this information. Additionally, X-ray radiographic techniques, like X-ray diffraction (XRD), facilitate investigations into the crystallographic properties associated with the construct’s structure. Diagnostic methodologies incorporating microscopy, notably micro-computed tomography (µCT), become instrumental in verifying the capability of bio-inspired mineralised scaffolds to enable in vitro investigations of cell–matrix interactions and their temporal dynamics concerning physiological bone remodelling. When these bio-inspired bone substitutes involve cellular seeding, especially with mesenchymal stem cells (MSCs) sourced from the patient’s bone marrow, the evaluation of construct efficacy becomes more intricate. Comprehensive tests are imperative to validate MSC differentiation into bone tissue cells (osteoblasts and osteocytes) and examine the distribution of newly formed bone tissue within the scaffold. Methods commonly employed to assess MSC differentiation include the alkaline phosphatase (ALP) assay, measuring ALP enzyme activity on osteoblast surfaces, and the calcium assay, quantifying calcium concentration within the construct. Immunohistochemistry and immunomicroscopy techniques further contribute to validating MSC differentiation and assessing the distribution of newly formed bone tissue. Molecules like osteopontin and type I collagen serve as indicators, providing information on bone regeneration based on their positioning relative to the extracellular matrix and cell nuclei. Diagnostic techniques such as picro-sirius red staining protocol for collagen (PSR) and alizarin red staining (AR) are employed to visualise collagen distribution and calcium distribution, respectively, within the scaffold [137,165]. While these state-of-the-art imaging techniques provide invaluable insights, they fall short in offering visual information on the interaction between scaffold and bone for certain scaffold types, such as silk fibroin. In addressing this limitation, synchrotron imaging, an innovative diagnostic technique, has emerged [25]. With its capability to produce high-resolution images, this technology facilitates clear differentiation between the scaffold and the newly formed bone tissue, enabling the generation of precise 3D models. This groundbreaking approach is pivotal for advancing bone regeneration strategies by enhancing the understanding of scaffold morphology and pore distribution, crucial for further advancements in 3D-printing methodologies for these constructs.

Secondly, the ***validation of the model’s ability to predict bone function recovery*** necessitates the evaluation of the mechanical properties of the bone, as it plays a pivotal role in comprehending the functionality of the construct. The mechanical characteristics of the scaffold are inherently tied to the material mechanical properties, which are scrutinised through stress–strain (or load–deformation) analyses using techniques like compression and torsion tests, dynamic mechanical analysis, or rheological measurements [176]. Various methods and equations have been employed to assess the self-repair quality of bio-inspired scaffolds. Cyclic strain, tension, compression, and torsion (mechanical tests) are typically applied to ascertain critical material properties, including strength and stiffness, before and after self-repair [26,200]. The healing efficiency (HE), a dimensionless value expressing the percentage or ratio of recovery of the mechanical property, is concurrently calculated. Although researchers are actively working on developing reliable validation methods for the HE parameter, insights into the material’s quality and functional state can be gleaned by monitoring the structural functionality and self-repair capacity of the construct [176]. Additionally, to evaluate the efficacy of a bone construct and study its in vitro remodelling, it is imperative to confirm the cytocompatibility of the materials used in the construction of the bio-inspired scaffold. Cytocompatibility tests, involving monocultures of mesenchymal stem cells or progenitor cells of osteoblasts, osteoclasts, and osteocytes, are conducted to estimate the trend of osteoclastic reabsorption and bone formation through diagnostic analyses like immunofluorescence or electron microscopy [137,141].

However, the validation of bone function recovery for bio-inspired scaffolds extends beyond in vitro models alone. Promising in vitro models must progress to in vivo testing on animal models to verify their efficacy on a living being before pre-clinical testing on humans. This involves testing on animal models with similar pathologies, such as osteoporosis or critical dimension fractures, to those suffered by the patients for whom the final product is intended [221]. A notable limitation of these efficacy verification procedures lies in the unavoidable sacrifice of animals to complete the analysis of the bio-inspired scaffold [158]. To address this ethical concern, researchers have endeavoured to develop in vitro models that closely mimic human physiology to limit the need for animal sacrifice [222].

To further reduce and optimise the use of animal models and generate effective in vitro models, the study of Basic Multicellular Units (BMUs) is employed. BMUs, discrete temporary anatomic structures of osteoblasts and osteoclasts, represent a human cell system working synergistically to remodel bone tissue through turnover [222]. Researchers leverage BMUs to increase translational power towards in vivo settings, providing valuable insights into bone remodelling and the presence of newly formed bone tissue [222,223,224]. The spatio-temporal continuum model derived from the study of BMUs enables the validation of newly formed bone tissue presence, offering a comprehensive understanding of ideal bone remodelling trends and the detection of anomalies in bone growth in patients or animal models [222].

## 6. Conclusions

In conclusion, this study addresses several key ***gaps*** in current research related to the limitations of traditional scaffold materials and fabrication techniques, particularly in the field of bone tissue engineering. One major gap lies in the mechanical properties of conventional scaffold materials, such as metals, ceramics, and synthetic polymers, which often fail to mimic the complex hierarchical structures and biomechanical behaviour of natural bone. These traditional materials can lack the necessary balance between strength, flexibility, and biodegradability, leading to complications such as poor integration with native bone tissue or inadequate mechanical support during the healing process.

Additionally, traditional fabrication techniques, such as casting and moulding, are limited in their ability to create scaffolds with fine-tuned, porous architectures that are crucial for promoting cell infiltration, nutrient diffusion, and vascularisation. These methods often result in scaffolds with less precise control over internal geometries, which can impede the homogeneous differentiation of mesenchymal stem cells (MSCs) and the formation of new bone tissue.

This study introduces bio-inspired design principles, advanced digital fabrication methods (such as 3D printing), and innovative material combinations that address these gaps. By synergistically integrating advanced digital design with novel material choices, these scaffolds present themselves as a compelling alternative to traditional bone repair methods, addressing inherent limitations such as insufficient mechanical properties and poor biocompatibility.

These scaffolds not only provide essential structural support but also create an optimised three-dimensional environment conducive to cellular proliferation and differentiation, particularly in guiding mesenchymal stem cells (MSCs) toward osteogenic lineages, resulting in the formation of bone cells such as osteoblasts. Moreover, the versatility and adaptability of these scaffolds facilitate the incorporation of engineered features that enable the controlled release of growth factors and signalling molecules, thereby augmenting their effectiveness in promoting bone regeneration.

Distinct from conventional methodologies, bio-inspired scaffolds offer a secure and efficient alternative, characterised by enhanced biocompatibility and gradual biodegradation that align seamlessly with the body’s natural healing processes. The design philosophy draws heavily from the intricate porosity found in natural bone, a defining feature that is critical for achieving successful functional outcomes in regenerated bone tissue.

Material selection for these scaffolds necessitates a judicious consideration of bio-derived synthetic and natural polymers, with attention to mechanical properties, degradation kinetics, and overall biocompatibility. This thoughtful approach contributes significantly to the development of effective bio-inspired bone substitutes that not only mimic the structure of natural bone but also promote physiological compatibility.

The hierarchical and precisely controlled architecture of bio-inspired scaffolds is essential for replicating the nuanced mechanical properties of bone tissue, thereby emphasising the interplay between form and function across multiple scales. This integration of sophisticated design with advanced materials effectively addresses the growing demand for high-performance biomaterials and innovative digital fabrication techniques, fostering a harmonious relationship between technological advancement and biological sustainability in regenerative medicine.

In the context of healthcare, the sustainability of bio-derived materials presents a compelling alternative to conventional materials, particularly in the face of increasing environmental concerns. Bio-derived materials, such as silk fibroin, chitosan, and PCL, are often sourced from renewable resources and exhibit biodegradable properties, minimising long-term environmental impact after their use in medical applications. In contrast, conventional materials, such as synthetic polymers and metals, typically involve petroleum-based production processes and may contribute to plastic waste and pollution. As the healthcare industry shifts towards environmentally conscious solutions, leveraging bio-derived materials not only enhances biocompatibility and functionality in tissue engineering, but also aligns with global sustainability goals, promoting a more responsible approach to material selection in medical applications.

## 7. Future Trends in Personalised Bone Repair

The prospective advancements in the development of biomimetic bone substitutes are multifaceted, converging towards a shared objective of enhancing the regulation of bone remodelling. Notably, two specific avenues within these emerging prospects warrant significant attention.

**Primarily**, the application of image-guided failure assessment analyses, complemented by multi-scale mechanical testing within synchrotron facilities, offers a promising route for creating detailed models capable of studying real-time interactions between scaffold architecture and bone formation. By combining high-resolution imaging techniques, such as phase-contrast micro-CT and X-ray diffraction, with mechanical testing, researchers can investigate how scaffolds influence the biomechanical environment of bone and assess scaffold degradation and bone growth simultaneously. This investigation serves the purpose of informing the fabrication of 3D bio-inspired scaffolds, enabling homogeneous differentiation of mesenchymal stem cells (MSCs) into osteoblasts and osteocytes throughout the scaffold volume. Understanding how the hierarchical structure and porosity of scaffolds contribute to cell behaviour can lead to the development of scaffolds that mimic the natural bone’s mechanical and biological environment more accurately. Furthermore, neural networks (NNs), trained on high-resolution imaging data of bone and polymeric scaffolds, hold potential in capturing and predicting the transformation of MSCs into bone tissue cells within bioreactors, enabling personalised optimisation of scaffold design based on patient-specific data. These AI-driven approaches could revolutionise scaffold development by streamlining the complex process of bone tissue regeneration and reducing the need for extensive experimental trials.

**Secondly**, the integration of biocompatible sensors within scaffold architecture presents a potential strategy for exerting enhanced control over bone remodelling processes, a concept explored primarily in metallic scaffolds to date. The concept of “smart scaffolds” introduces embedded sensors designed to detect alterations in mechanical properties and chemical cues within scaffold-based tissue-engineered constructs [67,225]. These sensors, such as strain gauges or electrochemical sensors, can provide real-time data on cellular activities, including proliferation, differentiation, and bone regeneration. For example, 3D-printed smart scaffolds [226] incorporating integrated sensors could monitor in vitro MSC behaviour, ensuring optimal conditions for osteogenesis by adjusting parameters such as nutrient supply or mechanical stimulation within bioreactors [227,228,229,230]. In conventional bioreactors, where imaging techniques may risk sample contamination, these sensors offer a contamination-free alternative to monitor scaffold–cell interactions.

Moreover, the application of these sensors extends to in vivo scenarios, where they can monitor the bone remodelling process within the body, providing valuable insights into scaffold integration, remodelling rates, and scaffold biodegradation. Recent strides in developing biodegradable implantable sensors, capable of monitoring diverse biological parameters such as oxygen, pressure, and glucose levels [231], underscore the transformative potential of such technology. For instance, biocompatible pressure sensors, exemplified by a 3D-printed scaffold functionalised with PEDOT (poly(3,4-ethylenedioxythiophene) polystyrene sulfonate) [232], exhibit noteworthy sensitivity and could be instrumental in assessing the mechanical environment surrounding bone defects. This capability extends to monitoring pressure fluctuations, tissue stiffness, or even the mechanical integrity of scaffolds under load-bearing conditions. By providing real-time feedback on biological processes, these sensors can inform personalised treatment plans, enabling tailored adjustments to scaffold design, patient care, and rehabilitation strategies. The diverse range of sensors and scaffold designs explored in recent studies highlights the versatility and adaptability of this approach. With the continued integration of advanced materials and sensor technologies, the potential for “smart” biomimetic scaffolds to revolutionise precision medicine becomes increasingly apparent. These innovations open new avenues for precision and efficiency in the realm of regenerative medicine, particularly in developing adaptive, real-time monitored scaffolds that respond dynamically to physiological changes, ensuring more efficient and effective healing outcomes.

Moving forward, future research directions should explore the full integration of machine learning algorithms for real-time scaffold optimisation, incorporating data from embedded sensors to continuously refine scaffold properties in response to changing biological conditions. Additionally, advancements in biodegradable sensor materials and wireless transmission technologies will further extend the applicability of smart scaffolds in both in vitro and in vivo environments, offering new possibilities for patient-specific regenerative therapies. Through a combination of real-time monitoring, AI-driven design, and sensor integration, these developments stand to significantly enhance the scope of personalised medicine in bone tissue engineering.

## Figures and Tables

**Table 2:** Advantages and limitations associated with distinct 3D-printing methodologies.

3D-Printing Techniques	Resolution	Pros	Cons	Ref.
Based on extrusion or drawing such as direct inkjet, bio-ink, and electrospinning printing.	Extrusion: 50–500 µm; Direct inkjet: 120–470 µm; Bio-ink: 100–1000 µm; Electrospinning: 50–500 nm.	Widely used for printing polymer scaffolds (such as PCL and PLA).	Limited by the shape and diametre of the nozzle. Restricted use due to its ability to effectively extrude material.	[182,183,184]
Based on photopolymerisation such as stereolithography, digital light processing, and two-photon polymerisation techniques.	Stereolithography: 25–150 µm; Digital light processing: 50–100 µm; Two-photon polymerisation: <1 µm.	Creation of patient-specific bio-inspired scaffolds: permit the material to take any shape.	Limited to photosensitive polymers.	[182,185,186]
Selective sintering, melting, or fusion techniques that use different energy sources (ion beam, laser, electron beam, or thermal sintering).	Selective sintering: 100–300 µm; Ion beam selecting melting: 10–100 µm; Laser (SLS): 30–200 µm; Electron beam (EBM): 100–200 µm; Thermal sintering: 100–300 µm.	Allow a high degree of control over the final shape of the scaffold.	Limited by the high cost of energy sources. This limit can be overcome by using the binder jet technique, which replaces the energy source with a liquid binder such as water or phosphoric acid.	[187,188]
